# Rapid itch relief with the biologic agent nemolizumab in Atopic dermatitis and Prurigo nodularis: Promise, complexity and clinical implications

**DOI:** 10.1111/jdv.70389

**Published:** 2026-04-25

**Authors:** Karoline Lukaschek, Elke Weisshaar

**Affiliations:** ^1^ Division of Occupational Dermatology, Department of Dermatology Ruprecht‐Karls University Heidelberg Heidelberg Germany

Chronic itch (CI) is among the most distressing symptoms for patients with Atopic dermatitis (AD) and Prurigo nodularis (PN), profoundly affecting sleep, daily functioning and quality of life. In this context, nemolizumab represents an important therapeutic advance. By offering rapid and clinically meaningful itch reduction, it substantially enriches the dermatological treatment landscape and directly addresses a symptom that patients consistently identify as their highest therapeutic priority.

The post hoc analysis by Staender et al. of the pivotal phase 3 trials—ARCADIA 1 and ARCADIA 2 in AD (*N* = 1728), and OLYMPIA 1 and OLYMPIA 2 in PN (*N* = 560)—provides a focused and clinically appealing perspective on the very early course of itch and sleep disturbance following treatment initiation.^1^ Drawing on these large, well‐conducted randomized controlled trials, which include validated patient‐reported outcome measures, the authors demonstrate a consistent separation from placebo as early as Day 2. The concordance of findings across both disease entities and multiple studies strengthens confidence that rapid itch reduction is a clinically relevant finding observed with nemolizumab treatment.

From a clinical standpoint, early itch relief is highly relevant, especially to reduce the itch‐scratch cycle, and even modest improvements within the first days of treatment may improve sleep, daytime functioning and psychological well‐being, support adherence and facilitate shared decision‐making, particularly in patients with severe symptom burden. In this regard, nemolizumab represents a valuable addition to current therapeutic options. However, itch in chronic inflammatory skin diseases is biologically complex and cannot be attributed to a single pathway alone. While interleukin‐31 is a key mediator of itch, especially CI, itch arises from a broader neuroimmune network involving multiple cytokines, immune cells and neural circuits.^2^ Consequently, rapid itch relief through IL‐31 blockade should be understood as targeting one important axis within a multifactorial process, rather than as a comprehensive solution for itch across all patients. This biological complexity and pathophysiology may partly explain interindividual variability in response and underscores the need for personalized treatment strategies.

Given the post hoc design of the analysis and the not pre‐specified early time points, the findings should be interpreted as exploratory with regard to early‐onset efficacy. In line with this post hoc focus, the analysis does not further address adverse events beyond those reported in the original phase 3 trials. Interpretation of early efficacy signals should therefore consider individual tolerability and adverse event profiles in routine clinical practice.

For the practising clinician, these findings highlight an increasingly important challenge: deciding which cytokine‐targeted pathway is most appropriate for an individual patient. With several interleukin‐directed therapies available,^3^ each modulating distinct inflammatory mechanisms, treatment selection requires careful alignment of biological rationale with dominant clinical features.^4^ Rapid itch relief may be decisive for some patients, while others may prioritize broader disease control, long‐term outcomes or individual tolerability. Evidence that helps to distinguish which patients are most likely to benefit from specific cytokine blockade is therefore of substantial practical relevance.

Finally, as is common in contemporary phase 3 dermatology research, both the underlying trials and the present analysis were industry sponsored. While this is standard in drug development and does not detract from the quality of the data, post hoc findings warrant careful interpretation.

In summary, nemolizumab is an important and welcome addition to current treatment options for patients with AD and PN, offering rapid and meaningful itch reduction. When interpreted in light of the multifactorial pathophysiology and origin of itch^4^ and the post hoc nature of the analysis, the findings provide valuable clinical insight while reinforcing the need for nuanced, patient‐centred treatment decisions. Long‐term extension studies suggest sustained disease control with nemolizumab.^5^ Future research should investigate long‐term control of CI with nemolizumab and its role in multimodal treatment of AD and PN.

## CONFLICT OF INTEREST STATEMENT

KL: None. EW: Advisory board: Galderma, Leo, Sanofi; Clinical studies: Galderma, Incyte, Celldex.

## Data Availability

Data sharing is not applicable to this article as no new data were created or analysed in this study.
